# A Structure–Function Dataset With Known Rates of Glaucomatous Progression

**DOI:** 10.1167/tvst.15.2.28

**Published:** 2026-02-25

**Authors:** Andrew Turpin, Vasanth Muthusamy, Munis Raviselvan, Allison M McKendrick

**Affiliations:** 1 Lions Eye Institute, Perth, Australia; 2 School of Population Health, Curtin University, Perth, Australia; 3 Department of Optometry & Vision Science, University of Western Australia, Perth, Australia

**Keywords:** dataset, glaucoma progression, structure–function, cpRNFL, visual fields

## Abstract

**Purpose:**

This article describes the Lions Eye Institute Structure–Function Dataset (LEI-SFD), an open, synthetic dataset of both structural and functional measurements from eyes with glaucoma that are progressing at known rates.

**Methods:**

The LEI-SFD contains static automated perimetry (SAP) and retinal nerve fiber layer (RNFL) thickness data (circumpapillary RNFL [cpRNFL]) that is progressing at known rates for 10 visits over 5 years. Measurements at the 10th visit and progression rates are taken from curated clinical data collected at Lions Eye Institute, Perth, Australia. Measurement noise is added based on existing literature.

**Results:**

The dataset contains 162 eyes with a mean (SD) baseline mean deviation of −2.6 (4.0) dB (minimum, −17.6; maximum, 1.6) and a mean (SD) cpRNFL thickness of 77.7 (13.5) microns (minimum, 43; maximum, 109). The average number of progressing SAP locations per eye is 20.8, with a mean pointwise rate of −0.6 dB/y. Using Permutation of Pointwise Linear Regression (PoPLR) to assess progression on the resulting datasets gives similar classification results to those published on clinical data.

**Conclusions:**

This open dataset contains longitudinal, linked structural and functional data with known progression rates. By using visit data and progression rates from real eyes to seed its synthetic generation, relationships between structure and function in current clinical data should be preserved, but with ground-truth progression rates being known.

**Translational Relevance:**

This open dataset will allow the assessment of the performance of methods for determining glaucomatous progression that use both structure and function on a common benchmark.

## Introduction

Improving the detection of glaucomatous progression is an important clinical problem for routine care and for optimizing clinical trials and hence is an active area of research. At the time of writing, glaucoma care involves the measurement of structural parameters deemed relevant to glaucoma, typically using optical coherence tomography (OCT), in addition to the measurement of visual function using perimetry. Most methods for detecting progression and measuring its rate of change have concentrated on either structural or functional data alone. There are some exceptions, that is, methods for detecting progression that aim to combine structural and functional data.^1^^–^^3^ Developing and evaluating the performance of these methods requires a time series of several years of linked structural and functional data from eyes with glaucoma. Methods to date have been derived and tested on datasets that are not readily available for others to use and therefore differ between studies. Moreover, given that the datasets are empirical, the true rate of progression is conflated with various forms of measurement noise; hence, the underlying true rate is unknown. This is the case regardless of whether the data were collected as part of a clinical trial or under the more variable conditions of routine clinical glaucoma care.

The dataset described here was designed to create an open-access benchmark dataset where the ground truth of true progression rates in a treated glaucoma population is known, so that it can be used to compare the performance of methods for progression detection and to support the development of new prognosis monitoring methods. The dataset, which we name the Lions Eye Institute Structure–Function Dataset (LEI-SFD), has defined ground-truth progression rates in both visual field (52 locations in the Humphrey Field Analyzer 24–2 pattern) and OCT measurements (six circumpapillary retinal nerve fiber layer [cpRNFL] thickness sector values collected on a Spectralis OCT, Heidelberg Engineering, referred to as “sectors” throughout) in the same eyes. The progression rates and final measurements for each eye were derived from empirical data collected during routine glaucoma care at the Lions Eye Institute, Perth, Australia. The measurements for visits 1 to *n* – 1 preceding the final visit *n* were artificially derived by “anti-progressing” from the final empirical measurement *n* back in time at the given rates for an eye. That is, measurements for visit *n* – 1 are derived by subtracting the true rate of progression for each measurement modality from visit *n* and so on. This method has been used previously for deriving artificial longitudinal visual field sequences that preserve spatial clinical features.^4^^,^^5^

## Methods

**Figure 1.**
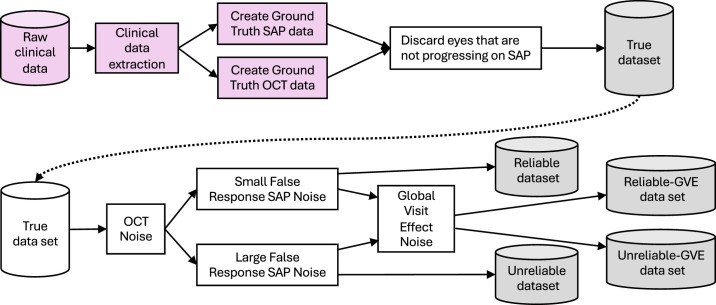
Overall process for generating the five base datasets of progressing data that make up the LEI-SFD. *Pink* indicates use of clinical data.

Overall, the process followed to construct the dataset consisted of five steps, summarized in Figure 1:

1. Extract clinical data for eyes with glaucoma.
2. Create ground-truth OCT data from the Spectralis clinical data.
3. Create ground-truth static automated perimetry data from the Humphrey Field Analyzer (HFA) clinical data.
4. Add OCT noise to the ground truth to get synthetic measured OCT data.
5. Add a variety of visual field (VF) noise to the ground truth to get synthetic measured VF data.

We discuss each of these steps in the following subsections.

### Clinical Data Extraction

**Figure 2.**
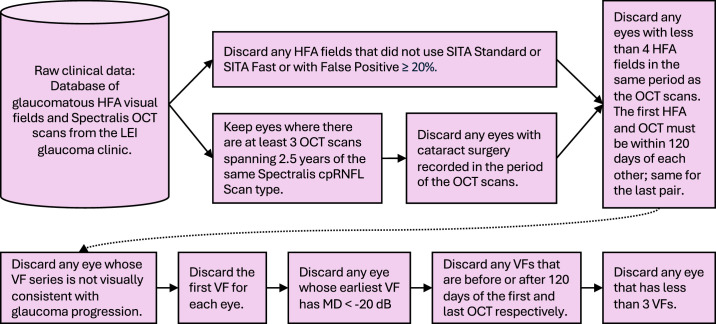
Procedure for collecting clinical data for input into data generation. *Pink* indicates use of clinical data.

Using a combination of electronic data filtering and manual inspection, records from people with ophthalmologic diagnoses of glaucoma attending the Lions Eye Institute (Perth, Australia) were collected using the method in Figure 2.

Potential eyes for inclusion in the initial pool of data were required to meet the following criteria:

• Spherical equivalent refractive error no less than −6 diopters (D)
• No retinal pathologies affecting visual field estimates except glaucoma
• No cataract surgery performed within the visual field series
• Diagnosis of glaucoma or glaucoma suspect with a likely pattern of glaucomatous progression on visual inspection of HFA data. Note rates of progression are checked numerically in the processes for extracting ground truth described in subsequent sections.

**Figure 3.**
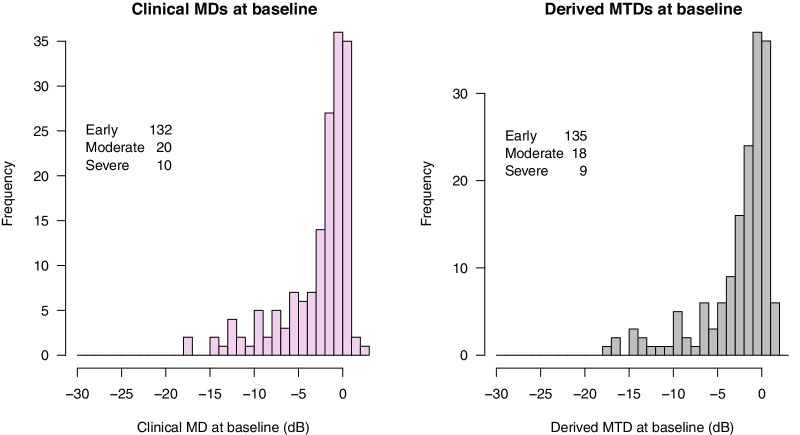
Mean deviations (MDs) at baseline in the clinical data (*pink*, *left*) and mean total deviation in the derived data (*gray*, *right*). Early is MD > −6 dB, moderate −12 ≤ MD < −6 dB, and severe < −12 dB.

At the beginning of the workflow in Figure 2, we had 220 eyes with suitable OCT scans and HFA fields in the same period with the diagnosis of glaucoma and no relevant comorbidities, as determined from chart review. All were undergoing treatment and monitoring of differing levels, as is typical of routine clinical care. After applying the logic in the figure, we had 162 eyes of 114 people remaining. Table 1 describes the composition of the clinical data used to seed the derived data, and Table 2 gives descriptive statistics of the HFA and OCT for both the clinical and derived datasets. Figure 3 shows a histogram of HFA 24–2 mean deviations at baseline.

**Table 1.** Summary of the Clinical Data Used to Seed Generation of the Synthetic Data, Including Diagnosis at the Final Visit and Any Surgeries Performed Before the Final Visit

Attribute	Mean	SD
Number of female eyes (59 people)	85	—
Number of male eyes (55 people)	77	—
Age at baseline, y	62.5	11.3
Age at final, y	67.7	11.5
Diagnosis		Number

Glaucoma suspect		29
Normal-tension glaucoma		11
POAG		99
PACG		15
Pigment dispersion glaucoma		5
Pseudoexfoliation glaucoma		3
Surgeries		Number

Glaucoma surgery		23
Minimally invasive glaucoma surgery		9
Peripheral iridotomy		16
SLT		30

PACG, primary angle closure glaucoma; POAG, primary open angle glaucoma; SLT, selective laser trabeculoplasty.

**Table 2.** Summary Statistics for the HFA and OCT of the Clinical Dataset Used to Seed Generation of the Derived Data and the Final Derived Data

	Clinical Data				Derived Data			
Attribute	Mean	SD	Median	Minimum, Maximum	Mean	SD	Median	Minimum, Maximum
HFA per eye								
Number of tests	6.9	2.2	7	4, 14	10	—	—	—
Gap between each test (days)^*^	327	180	315	0, 1449	183	—	—	—
Span of tests (years)	5.3	1.4	5.1	2.3, 10.7	5	—	—	—
MD at baseline (dB)	−2.7	4	−1.2	−17.1, 2.2	−2.6	4	−1.2	−17.6, 1.6
MD at final (dB)	−3.3	4.7	−1.6	−21.3, 1.9	−3.7	4.8	−2	−21.4, 1.5
MD slopes <0 (dB/y)	−0.3	0.4	−0.2	−2.9, −0.005	−0.2	0.3	−0.1	−2.1, −0.001
All pointwise slopes <0 (dB/y)	—	—	—	—	−0.2	0.3	−0.1	−2.1, −0.001
Minimum pointwise slope (dB/y)	—	—	—	—	−1.7	1.5	−1	−6.6, −0.07
Number of progressing locations	—	—	—	—	20.8	12.3	20	1.0, 52
OCT per eye								
Number of tests	5.4	1.8	5	3.0, 12	10	—	—	—
Gap between each test (days)^†^	436.1	316.3	368	0.0, 3731	183	—	—	—
Span of tests (years)	5.3	1.4	5.1	2.6, 10.7	5	—	—	—
Quality score	24.8	4.2	25	15.0, 37	—	—	—	—
Global baseline (microns)	79.6	14.1	80.5	44, 112	77.7	13.5	79	43, 109
Global final (microns)	76.2	14.8	78	36, 107	75.2	13.9	76	38, 105
T sector at baseline (microns)	62.2	13.4	61	29, 101	61.7	13.1	60	29, 102
TS sector at baseline (microns)	106.6	27.4	104	40, 166	105.4	27.2	103	43, 159
NS sector at baseline (microns)	87.8	23.4	86	38, 162	87.3	22.7	86	38, 157
N sector at baseline (microns)	64.6	12.5	64	31, 97	64	12.5	63	32, 95
NI sector at baseline (microns)	86.9	22.3	84.5	42, 156	86.3	22.1	84	44, 156
TI sector at baseline (microns)	106	34.6	114	33, 171	104.9	34.3	113	29, 171
T sector at final (microns)	59.8	13.2	59	30, 97	59.9	13.1	59	29, 102
TS sector at final (microns)	101.2	28.8	101.5	38, 152	101.6	29	101	39, 154
NS sector at final (microns)	85	23.1	83	34, 146	85.2	22.6	84	35, 146
N sector at final (microns)	62.1	12.9	62	33, 95	62.3	12.4	62	32, 95
NI sector at final (microns/y)	83.3	22.6	80	38, 156	83.4	22.7	81	38, 156
TI sector at final (microns/y)	99.8	35.4	101	24, 162	100.2	35.9	102	24, 171
T sector slope (microns/y)	−0.5	0.9	−0.4	−4.1, 1.9	−0.4	0.6	0	−3.2, 0
TS sector slope (microns/y)	−1	1.8	−0.6	−8.9, 2.7	−0.8	1.4	0	−8.0, 0
NS sector slope (microns/y)	−0.5	1.4	−0.4	−9.3, 2.2	−0.4	1	0	−8.3, 0
N sector slope (microns/y)	−0.4	0.9	−0.5	−3.3, 2.6	−0.3	0.6	0	−3.0, 0
NI sector slope (microns/y)	−0.6	1.3	−0.5	−6.4, 3.5	−0.6	1	0	−5.8, 0
TI sector slope (microns/y)	−1.1	2.1	−0.6	−13.3, 3.8	−0.9	1.7	0	−12.0, 0

MD, mean deviation; N, nasal; NI, nasal inferior; NS, nasal superior; T, temporal; TI, temporal inferioe; TS, temporal superior.

* Gap of 0 days indicates repeated tests for two eyes, both of which had at least six unique dates of VF tests.

† Gap of 0 days indicates repeated tests for five eyes, all of which had at least three unique dates of OCT tests.

### Deriving OCT Ground Truth

**Figure 4.**
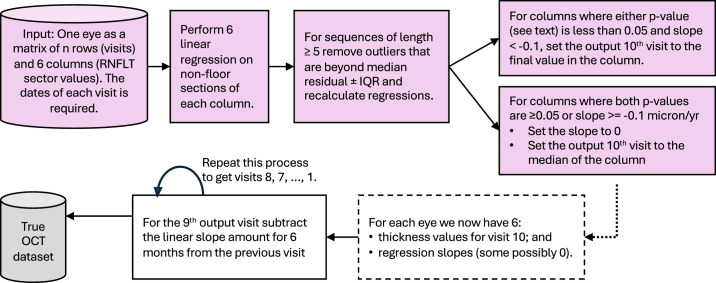
Process for finding progression slopes for each pixel in the series of OCT visits for one eye and then generating nine artificial visits backward from the final clinical visit. *Pink* indicates use of clinical data.

The image quality and automated segmentation of each Spectralis OCT image were manually checked and corrected if required (author VM), and the 3.5-mm ring cpRNFL thickness values were exported as a matrix of six thickness values (sectors) with one row per visit. All scans had a quality score of 15 or higher, which is the manufacturer’s recommended minimum scan quality.^6^ This was fed into the process outlined in Figure 4.

In an aim to only include progressing slopes that were likely to be genuine, we only included negative slopes less than −0.1 microns/y, where the *P* value of either a *t*-test or permutation test that the slope was zero was less than 0.05 (after removing outliers for sequences of length 5 or more). A sensitivity and specificity analysis to justify these choices is included in the Appendix. From this process, we end up with “True” cpRNFL thickness data that has six sectors per visit and 10 visits per eye. Note there is no correction for aging in this process, as it is likely to be minimal over a 5-year period at a sector level, having previously been reported to be −0.2 microns per year globally.^7^ It is also possible that some eyes have all sectors with no progression.

### Deriving Ground-Truth HFA Data

**Figure 5.**
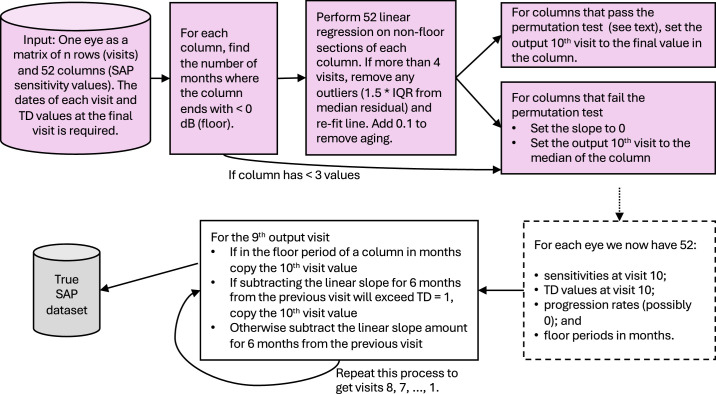
Process for finding progression slopes for each location in the series of static automated perimetry visits for one eye and then generating nine artificial visits backward from the final clinical visit. *Pink* indicates use of clinical data.

The HFA thresholds and total deviation values were obtained for each visit in the data remaining from Figure 2. These were then fed into the process outlined in Figure 5. Fitted linear regression slopes for sensitivity at each location in an eye over the visits available were included if they passed one of two tests.

The first check was a combination of a check that the slope of a linear regression with outliers removed (median residuals ± 1.5 interquartile range) was less than that given in the following table and that the *P* value returned on a permutation test on the slope (null hypothesis slope = 0) was less than 0.5.

**Table:** 


Series length	4	5	6	7	8	≥9
Slope cutoff	−1.6	−1.1	−0.9	−0.7	−0.5	−0.4

The seemingly generous *P* value cutoff catches the locations experiencing a very rapid progression over a subsection of the series (“an event”) and, when combined with the slope cutoffs, also catches locations decreasing at a roughly linear rate that would most likely be considered progression while keeping specificity at 99% (see the Appendix for details).

The second test was for locations with negative slopes that failed the first test. These locations were included as progressing if they had a negative slope (note aging has been removed), a *P* value from the permutation test <0.5, and at least one neighbor that passed either test. The rationale here is that locations with small negative slopes are hard to definitively call progressing in isolation, but a cluster or chain of spatially collected locations with small negative slopes (and *P* values <0.5), or locations with small negative slopes that spatially bridge between definitely progressing locations, are more likely to be progressing themselves. Note the specificity for these added locations is still greater than 95%, as the chance of erroneously choosing two neighboring stable locations is at about 0.01 * 0.50.

During the antiprogression calculations, if the total deviation (TD) of a location exceeded zero, it was not subject to further antiprogression. Note that both raw threshold and TD values were computed in this process, and both are included in the final datasets. We computed slopes on the raw dB values so that the floor effect could be easily identified, and 0.1 dB/y was added to slopes to “reverse” aging effects present in the clinical data.

### Adding Noise to OCT Data

**Figure 6.**
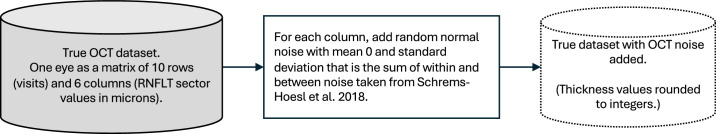
Process for adding noise to True OCT data.

From the True dataset of 162 eyes, we proceeded to derive measured OCT by adding noise to each 10 × 6 matrix of values for each eye, as outlined in Figure 6. The within- and between-visit OCT noise, taken from Schrems-Hoesl et al.,^8^ is shown in Table 3.

**Table 3.** Standard Deviation of Normal Noise Added to Each Sector

Characteristic	Temporal	Temporal Superior	Nasal Superior	Nasal	Nasal Inferior	Temporal Inferior
First visit	4.18	5.18	4.56	5.53	5.97	3.23
Subsequent visits	3.11	3.5	3.03	3.55	3.81	2.43

### Adding Noise to True Visual Field Data

**Figure 7.**
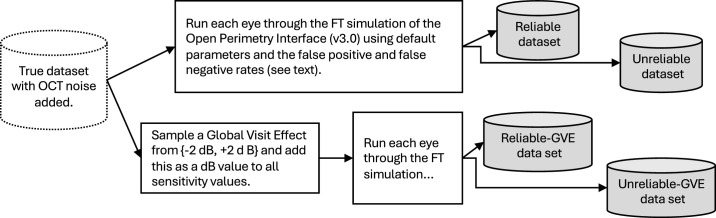
Process for adding visual field measurement noise to the True static automated perimetry values to get the final datasets.

Perimetric noise was introduced by simulating measurements of True data using the Full Threshold (FT, a 4–2 staircase) algorithm, as implemented in the Open Perimetry Interface (R package OPI, v3.0)^9^ and using the “SimHenson” mode of simulation in the OPI with default parameters and different false-positive and false-negative response rates (Fig. 7). These simulations mimic operation of the FT algorithm at every location in the visual field, and thus noise is being added at each location both through stochastic seen/unseen responses to stimuli and through the spatial and decision logic of the FT algorithm. Note that the noise is similar, by design, to the SITA Standard algorithm^10^ that is currently in common use. In addition, for some instances, a value was added to true values prior to simulation as a “general height” change or “global visit effect (GVE).” This produces four different datasets described in Table 4. Each of these four have the same underlying true visual field and OCT values, but different random seeds (1, 2, 3, and 4) are used for the generation of the random noise for each set.

**Table 4.** The Four Datasets Produced With Different Noise Conditions for the Visual Field Data

Dataset Name	False-Positive Rate	False-Negative Rate	GVE
Reliable	3%	1%	—
Unreliable	15%	3%	—
Reliable_gve	3%	1%	For every sequence, randomly select one visit with equal probability and add ±2 dB to True with equal chance before simulating the Full Threshold.
Unreliable_gve	15%	3%	As above

### Generation of Stable Datasets

In addition to the four progressing datasets, we also generated four stable datasets by simply repeating the 10th visit from the progressing eye 10 times and using that as the true underlying values for both visual field and OCT. We followed the same process for adding noise to the stable datasets as for the progressing datasets but used random seeds 5, 6, 7, and 8.

### Location and License

The dataset and accompanying code are published under the BSD 3-Clause license on GitHub at https://github.com/Lions-Eye-Institute/LEI_SFD.

## Data Summary

**Figure 8.**
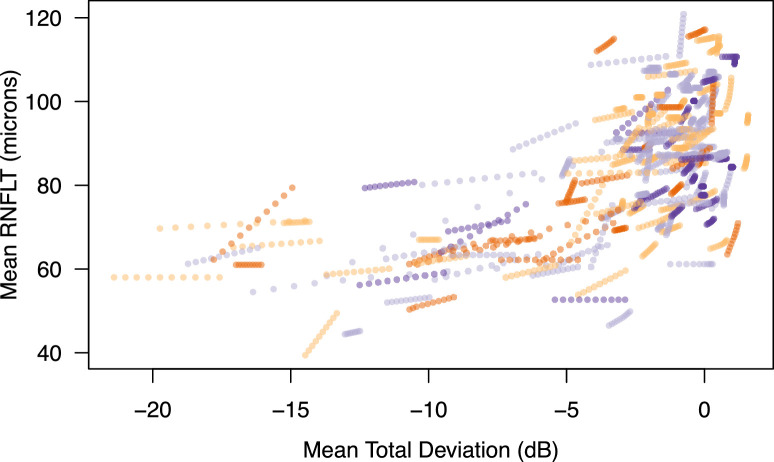
For each eye, the mean TD versus mean RNFLT for the 10 visits in the True dataset is plotted. Each eye has a consistent but not unique color; thus, “strings” of 10 dots forming a curve/line of the same color are for one eye.

Figure 8 shows a summary of the change in mean TD and average RNFLT for each eye over the 10 visits in the True dataset. As can be seen, many of the mean TDs are near zero, and some are even positive, and thus the dataset contains many eyes that are progressing from normal or early glaucoma. Note also that while each eye is progressing pointwise or sector-wise on each dimension at a fixed linear rate, the mean progression can be nonlinear as points or sectors reach their relevant measurement floor and stop progressing.

**Figure 9.**
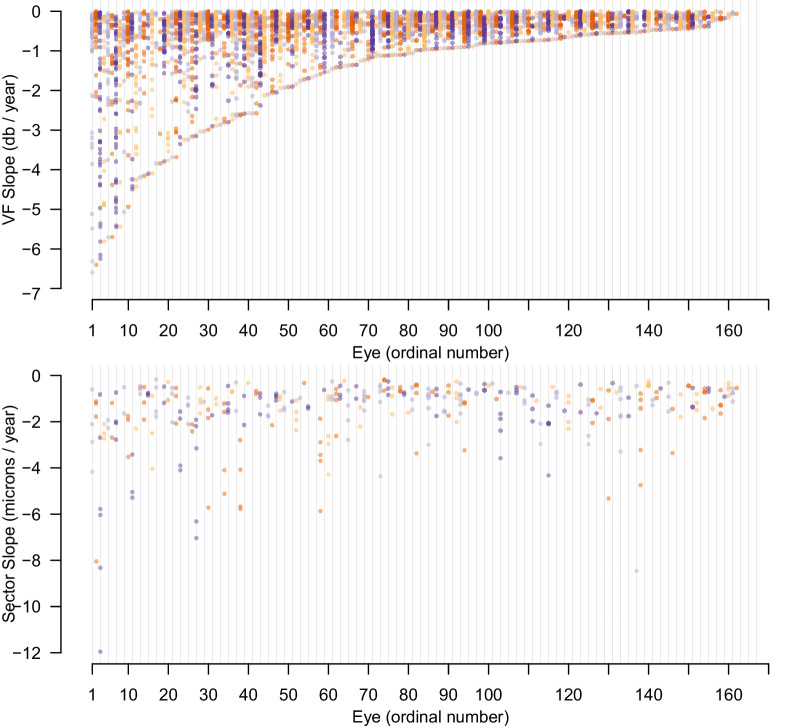
The number of progressing locations in the VF of each eye in the True dataset and the rate of change in those locations, sorted by minimum slope of progression (*top*) and sector slopes in the same eyes (*bottom*). Each eye is assigned its own color arbitrarily, so vertical columns of the same color are for the same eye. Vertical lines are shown every 23 eyes to aid orientation.

Figure 9 plots the slope of visual field progression in dB/y for all locations that are progressing for each eye in the True dataset. As can be seen, 81 eyes have at least one location progressing by more than −1 dB/y (left part of figure), and the remainder have a small number of visual field locations progressing by less than −1 dB/y.

Taken together, these two figures show that while the True dataset does contain progressing fields and OCT data, some of the rates of progression are small, and the baseline is often close to normal. Thus, this dataset provides a challenge for methods that aim to detect progression; once noise is added to form the Reliable and Unreliable datasets, small progression signals could be hard to detect. However, as these rates and final measurements are taken from contemporary, real patients, we believe that this dataset does represent a group of eyes that should be subject to being classified as progressing by analysis methods.

The sex and age at final HFA for each person are included in a separate file (person.csv).

### Technical Validation

**Figure 10.**
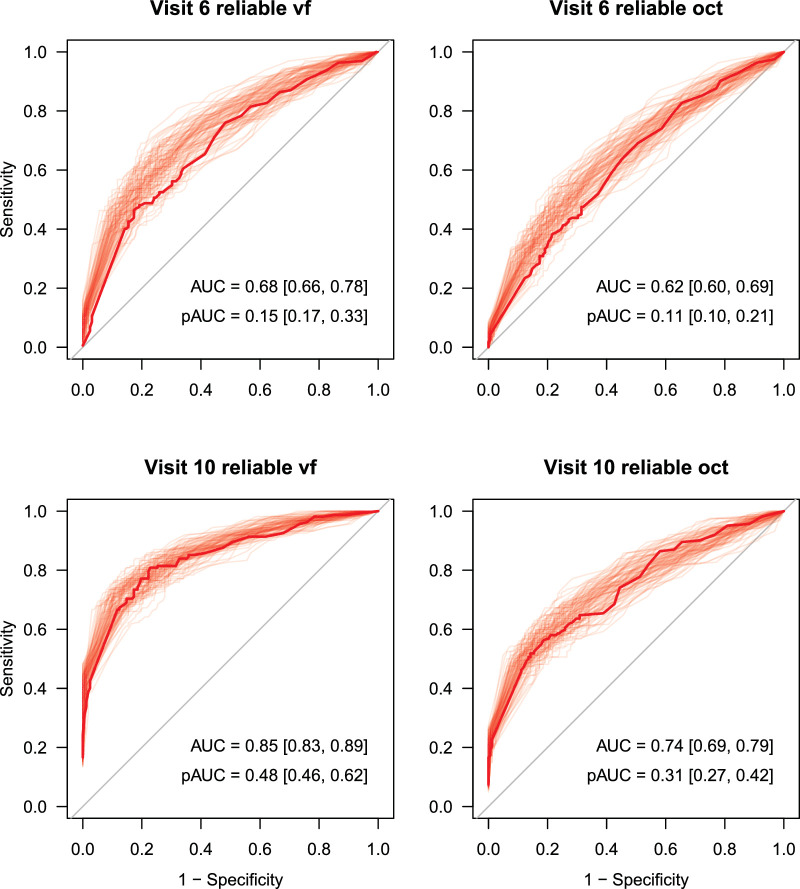
ROC curves for the detection of progression using PoPLR on both VF and OCT data separately at visits 6 and 10 for the Reliable datasets. The *solid curve* is for the dataset in LEI-SFD1, and the *light lines* represent 99 other datasets generated from the True dataset in LEI-SFD1 using the code supplied with the dataset (generate.r with random seeds).

As a simple benchmark for classifying these synthetic eyes as progressing (or not), we ran the Permutation of Pointwise Linear Regression (PoPLR) method^11^ to determine progression in the datasets. By varying the cutoff *P* value for calling progression, we constructed receiver operating characteristic (ROC) curves, as shown in Figure 10. The solid line in the figures represents the ROC for the LEI-SFD1 datasets, with the lighter lines representing 99 other datasets that we generated from True using the same code as for the base datasets, but using different random seeds to generate the VF and OCT noise. The code for generating datasets is provided at our GitHub site (https://github.com/Lions-Eye-Institute/LEI_SFD).

Table 5 shows all area under the curve (AUC) (as a percentage) and pAUC (computed for the portion of the curve where specificity ≤90% and shown as a percentage of 0.1) values for visits 6 through 10 on the standard derived datasets (LEI-SFD1). The stable datasets used to compute specificity were the same in both tables (taken from LEI-SFD1). These results are commensurate with those in the original PoPLR study on clinical visual field data (where hit rate and false-positive rate were used in lieu of a ground truth), where pAUC is 12, 22, and 41 for the fifth, eighth, and final visits, respectively (derived from Fig. 2 of O'Leary et al.^11^).

**Table 5.** AUC and pAUC (Specificity ≤90%) for the LEI-SFD1 Datasets at Visits 6 to 10 as Calculated by PoPLR

		AUC for Visits 6 to 10					pAUC for Visits 6 to 10				
Dataset	Data Type	6	7	8	9	10	6	7	8	9	10
Reliable	vf	67.9	73.6	77.5	81.6	84.6	14.7	25.8	37.4	40.3	47.8
Reliable_gve	vf	61.5	66.0	66.7	67.9	71.6	14.9	24.0	21.5	27.7	31.6
Unreliable	vf	69.6	73.4	76.9	81.9	83.2	20.7	28.0	33.8	37.7	45.0
Unreliable_gve	vf	66.3	71.0	72.9	73.5	77.2	15.7	23.8	26.7	26.3	27.0
Reliable	oct	62.3	68.3	71.5	73.6	73.7	11.3	19.0	21.7	27.0	31.2

## Discussion

We have constructed our first version of the LEI-SFD, a synthetic dataset with linked visual field and cpRNFL thickness values for 162 eyes that are progressing at a known rate but based closely on empirical clinical data. By using visit data and progression rates from real eyes to seed the synthetic generation of these data, it is hoped that relationships between structure and function that are found in current clinical data are preserved, with the added benefit that the ground-truth progression rates are known.

The key benefit of defining the rates of progression in this dataset is that methods that attempt to estimate rates of progression can now measure the accuracy and precision of those estimates. In the current literature, this cannot be done because there is no ground truth; usually, classification metrics such as sensitivity, specificity, and AUC are reported. Thus, we envisage this dataset being used to benchmark new methods that use both OCT and VF data for predicting rates of change (in one or both metrics).

If these datasets prove useful to the research community, we plan on adding more eyes in the future, hence the “1” in the name of the current datasets. The dataset and code for the generation of similar datasets using the True data as a baseline are available at GitHub (https://github.com/Lions-Eye-Institute/LEI_SFD). We have deliberately used the random seeds of 1 through 8 to generate LEI-SFD1 (four progressing and four stable sets) in the R random number generator so that if other datasets are generated from True with different seeds, it is unlikely that the same noise will be added. We recommend that the seeds less than 103 be reserved for future releases of data.

It is important to recognize that the dataset was derived from a specific treated clinical population tested within the glaucoma clinics at the Lions Eye Institute in Perth and hence may have features that are different from or not relevant to some other clinical settings. Glaucoma care in Australia is very similar to that in other Western countries, and the Lions Eye Institute is a tertiary center that attracts referrals from the greater Perth region. We do not have access to the specific ethnic makeup of the individuals, but the clinical population at the Lions Eye Institute is consistent with broader demographics of the greater Perth region.^12^ The latest Australia census (2021) reports 59.5% of people in Perth being born in Australia, with the next most common locations of birth being England (8%), New Zealand (2.8%), India (2.8%), South Africa (1.8%), and Malaysia (1.5%). Typical of the multicultural nature of Australian major cities, 24% of households reported using a non-English language at home.

In addition to the above demographic features of the dataset, an additional specific feature of this dataset is that it contains very few fast progressors. We consider this feature to be a strength in terms of using this dataset as a test for future development of analytical techniques to detect progression. The sensitivity and specificity for detecting catastrophic progression relative to stable eyes are likely very high for most plausible analytical methods. It is the slow progressors that are harder to capture. Nevertheless, if the intent is to derive methods that accurately predict the rate of progression, any future use of our dataset will be limited to testing predictions on slow to moderate progression rates only.

Also important to note is that all true progression of both visual field and OCT in the dataset is at a fixed rate (linear, allowing for floors in the respective measurements) based on linear regression fits to our underlying clinical data. It is entirely possible that the true progression characteristics of some of the clinical eyes were not linear at a fixed rate; for example, episodic or irregular rates might have occurred. By summarizing progression as linear in both modalities, we are assuming that any smoothing or altering of the “true” characteristics of the progression applies equally to both the visual field and OCT data, and so some of the temporal relationships between the structural and functional measurements will be preserved in the synthetic data for eyes with nonlinear progression. Note also that we opted for a conservative definition of progression at a VF location or OCT sector using a criterion with 99% specificity (as described in the Appendix). For example, sensitivity to detecting a −0.5 dB/y change using our criteria is 10% or less for four to six visits. Consequently, locations or sectors with small progression rates (assuming they exist) in short clinical series will not have been detected in the clinical data and hence will not be represented in the synthetic data.

There are several other publicly available datasets of visual field data and one that also contains OCT data. We cross-reference our field naming convention (“data dictionary”) with theirs in the following table, which may assist some readers.

**Table:** 

	Visual Field Source	Field Names	RNFL Data	Field Names	Size
LEI_SFD (this article)	52 locations of HFA 24–2, sensitivities, and total deviations	vf.1, vf.2, . . ., vf.52 td.1, . . ., td.52	6 sectors of cpRNFL scan, Spectralis	oct.T, oct.TS, . . ., oct.TI	162 eyes, 10 visits per eye
Rotterdam Longitudinal Glaucomatous Visual Field data^13^	54 locations of HFA 24–2, sensitivities, total deviations	X, Y, THRESHOLD, TOTAL_DEVIATION	—	—	260 eyes, at least 15 visits per eye
UWHVF^14^	54 locations of HFA 24–2, sensitivities, total deviations, and more	Sens_1, . . ., Sens_54 TD_1, . . ., TD_54	—	—	7248 eyes, between 2 and 5 visits per eye
GRAPE^15^	61 locations of Octopus 900 G pattern	0, 1, . . ., 60	4 sector and global average, CIRRUS HD-OCT 5000	Mean, S, N, I, T	263 eyes, between 3 and 9 visits per eye

## Appendix

### Sensitivity and Specificity of Progressing Location Selection in HFA Data

We assume a collection of VF locations that all begin at 30 dB and progress at known slopes of −3, −2, −1, −0.5, −0.2, −0.1, and 0 dB/y (1000 locations for each slope). We simulated measurements of these locations for 4 to 10 visits using the Full Threshold testing algorithm with a 15% false-positive rate and 3% false-negative rate using the OPI “SimHenson” mode (see main text for further details). For each method of calling progression, we counted the “hit rate” on nonzero slopes to determine sensitivity and on the 1000 zero slopes to determine specificity.

In addition to the locations that have true linear progression, we also included six locations that had very rapid, nonlinear progression from our clinical data. These received the same treatment, being taken as true and run through the FT algorithm 1000 times, and are shown in the left-hand column of Table A1.

**Table A1.** Hit Rates (as Percentages) of Two Methods for Calling Progression on Stable Locations (First Row), Linearly Progressing Locations (Rows 2 to 7), and Nonlinear Locations (Final 6 Rows)

	*t*-Test *P* < 0.01 Sequence Length					Permutation Test Outliers Removed Sequence Length				
	4	5	6	7	10	4	5	6	7	10
0 dB/y	0.2	0.1	1.1	0.8	1.0	0.9	0.8	1.3	1.1	1.2
−3 dB/y	4.2	12.0	31.0	68.3	91.9	66.5	90.7	96.0	98.9	100.0
−2 dB/y	2.6	7.5	18.9	31.6	87.4	45.2	69.3	81.2	92.7	99.8
−1 dB/y	1.9	4.3	8.1	14.2	45.8	15.5	30.8	41.8	60.0	90.4
−0.5 dB/y	0.3	0.7	2.7	3.2	13.6	4.1	6.0	10.3	17.0	47.9
−0.2 dB/y	0.3	0.1	1.3	0.6	2.4	1.4	2.8	1.9	3.4	8.5
−0.1 dB/y	0.2	0.3	0.6	0.6	1.0	1.7	1.8	1.5	1.9	2.5
30 29 20 −1 −1		5.6					100.0			
30 29 −1 −1 −1		0.1					100.0			
30 25 20 −1 −1		12.6					99.9			
30 25 10 10 10		9.4					100.0			
26 29 26 22 −1 3 −1				56.2					100.0	
28 31 31 30 22 −1 14				0.2					99.3	

First column shows linear rate of change (rows 1 to 7) or dB values of the location (rows 8 to 13).

We investigated several methods and combinations of methods, but we report only two here. The first calls a location progressing if the slope is <0 and the *P* value of a *t*-test on the slope is below 0.01. The second method first removes outliers from a linear fit (any values with residuals outside the median ±1.5 times the interquartile range of the residuals) and then calls a location progressing if a permutation test on the slope of the fit to the cleaned data has a *P* value less than 0.5 and the slope is less than −1.6, −1.1, −0.9, −0.7, −0.5, and −0.4 for sequences of length 4, 5, 6, 7, 8, and ≥9, respectively. Note that the generous *P* value cutoff of 0.5 for the permutation test ensures a sensitivity of close to 100% for the “event” progression but also degrades specificity to about 50% if it is not accompanied by the slope cutoffs based on the length of the sequence. These slope cutoff values were derived as the lower 1 percentile of the distribution of slopes for stable sequences (thus giving a specificity of approximately 99%).

The first five data columns of Table A1 demonstrate that using the *P* value of a *t*-test on slope has low sensitivity, rarely calling progression until sequences are long and slopes are steep. The *t*-test has low sensitivity for all of the rapid locations (final six rows). Contrarily, the approach based on the permutation test is very sensitive on the rapid locations, calling all of them in nearly all 1000 repeats, and also has significantly higher sensitivity than the *t*-test method on the smaller linear slopes. Importantly, the permutation test approach maintains specificity at about the same level as the *t*-test (∼1%).

### Sensitivity and Specificity for Progressing Sector Selection in the OCT Data

To test the sensitivity and specificity of different methods for calling progression of OCT sectors, we modeled different sequences of length 4 through 10 and rates of linear decline of 0, −0.2, −0.5, −1, −2, −3, and −4 microns per visit for the nasal-inferior sector, starting at a thickness of 139 microns at the first visit. Noise around these sequences was added using a normal distribution of mean 0 and a standard deviation of noise of 5.96 (the sum of between and within noise from Schrems-Hoesl et al.^8^).

Two methods are presented. The first is simply calling a location progressing if a *t*-test on slope has a *P* value less than 0.01. The second first fits a linear regression and records the *t*-test *P* value, then removes outliers (any point with a residual outside the range median residual ±1.5 * interquartile range) and performs a permutation test on a new fit to the possibly reduced data. The final call is then a combination of the slope being less than −0.1 microns/y and both *P* values being less than 0.05.

Table A2 shows that the sensitivity of the *t*-test is poor and the permutation test–based approach is superior. However, using the permutation test approach does give away some specificity for short sequences (first row of panel b in Table A2 is higher than row 1 in panel a). This modeling is done on the most variable sector (nasal-inferior) according to Schrems-Hoesl et al.,^8^ and so we are confident that other sectors would not suffer the same drop in specificity. The gains in sensitivity for small slopes and shorter sequences are large. Note that using a criterion based solely on the permutation test after removing outliers without considering the *t*-test *P* value leads to slightly reduced sensitivity for short sequences in these simulations with no improvement in specificity (results not shown).

**Table A2.** Hit Rates (As Percentages) of Two Methods for Calling Progression on Stable Sectors (First Row in Each Panel) and Linearly Progressing Sectors (Rows 2 to 7 in Each Panel)

(a) *t*-Test *P* < 0.01							
	*n* = 4	*n* = 5	*n* = 6	*n* = 7	*n* = 8	*n* = 9	*n* = 10
0	1.4	1.3	1.2	1.5	0.4	1.0	0.6
−0.2	1.1	1.5	1.3	1.0	0.3	1.6	2.1
−0.5	1.2	1.1	1.5	0.8	1.8	2.0	3.0
−1	0.6	1.9	2.4	2.5	4.3	5.2	9.8
−2	1.6	3.4	5.0	7.8	18.1	27.4	45.4
−3	1.3	6.3	10.8	23.7	41.5	64.2	82.8
−4	3.4	6.4	19.7	41.8	70.8	91.1	98.6
(b) Slope <−0.1 and (Permutation Test *P* < 0.05 OR *t*-Test *P* < 0.05)							

	*n* = 4	*n* = 5	*n* = 6	*n* = 7	*n* = 8	*n* = 9	*n* = 10

0	8.6	5.2	4.5	6.1	4.2	3.9	4.2
−0.2	12.4	7.1	7.1	6.6	6.7	8.0	10.1
−0.5	13.8	9.5	10.1	8.6	11.1	12.4	15.3
−1	15.9	11.8	14.2	17.6	24.7	32.1	39.9
−2	23.4	22.6	31.0	42.7	61.7	75.1	87.9
−3	34.4	34.6	53.5	71.2	87.3	96.0	99.5
−4	41.8	49.3	73.8	90.5	98.1	100.0	100.0

Values in column 1 show the linear rate of change in microns per year.

## References

[1] Medeiros FA, Leite MT, Zangwill LM, Weinreb RN. Combining structural and functional measurements to improve detection of glaucoma progression using Bayesian hierarchical models. *Invest Ophthalmol Vis Sci*. 2011; 52: 5794–5803. 21693614 10.1167/iovs.10-7111 PMC3176049

[2] Medeiros FA, Zangwill LM, Girkin CA, Liebmann JM, Weinreb RN. Combining structural and functional measurements to improve estimates of rates of glaucomatous progression. *Am J Ophthalmol*. 2012; 153(6): 1197–1205.e1191. 22317914 10.1016/j.ajo.2011.11.015 PMC3804258

[3] Russell RA, Malik R, Chauhan BC, Crabb DP, Garway-Heath DF. Improved estimates of visual field progression using Bayesian linear regression to integrate structural information in patients with ocular hypertension. *Invest Ophthalmol Vis Sci*. 2012; 53: 2760–2769. 22467579 10.1167/iovs.11-7976 PMC4632869

[4] Spry PG, Bates AB, Johnson CA, Chauhan BC. Simulation of longitudinal threshold visual field data. *Invest Ophthalmol Vis Sci*. 2000; 41(8): 2192–2200. 10892862

[5] Turpin A, Morgan WH, McKendrick AM. Improving spatial resolution and test times of visual field testing using ARREST. *Transl Vis Sci Technol*. 2018; 7(5): 35. 10.1167/tvst.7.5.35 PMC6213773 30402342

[6] Huang Y, Gangaputra S, Lee KE, et al. Signal quality assessment of retinal optical coherence tomography images. *Invest Ophthalmol Vis Sci*. 2012; 53(4): 2133–2141. 22427567 10.1167/iovs.11-8755 PMC3995569

[7] Chauhan BC, Vianna JR, Sharpe GP, et al. Differential effects of aging in the macular retinal layers, neuroretinal rim, and peripapillary retinal nerve fiber layer. *Ophthalmology*. 2020; 127(2): 177–185. 31668716 10.1016/j.ophtha.2019.09.013 PMC6982591

[8] Schrems-Hoesl LM, Schrems WA, Laemmer R, Kruse FE, Mardin CY. Precision of optic nerve head and retinal nerve fiber layer parameter measurements by spectral-domain optical coherence tomography. *J Glaucoma*. 2018; 27(5): 407–414. 29329141 10.1097/IJG.0000000000000875

[9] Turpin A, Artes PH, McKendrick AM. The Open Perimetry Interface: an enabling tool for clinical visual psychophysics. *J Vis*. 2012; 12(11): 22. 10.1167/12.11.22 23104815

[10] Bengtsson B, Heijl A. Evaluation of a new perimetric threshold strategy, SITA, in patients with manifest and suspect glaucoma. *Acta Ophthalmol Scand*. 1998; 76(3): 268–272. 9686835 10.1034/j.1600-0420.1998.760303.x

[11] O'Leary N, Chauhan BC, Artes PH. Visual field progression in glaucoma: estimating the overall significance of deterioration with permutation analyses of pointwise linear regression (PoPLR). *Invest Ophthalmol Vis Sci*. 2012; 53(11): 6776–6784. 22952123 10.1167/iovs.12-10049

[12] Australian Bureau of Statistics. Greater Perth 2021 Census All persons QuickStats. https://www.abs.gov.au/census/find-census-data/quickstats/2021/5GPER. Accessed January 16, 2026.

[13] Bryan SR, Vermeer KA, Eilers PHC, Lemij HG, Lesaffre EM. Robust and censored modelling and prediction of progression in glaucomatous visual fields. *Invest Ophthalmol Vis Sci*. 2013; 54(10): 6694–6670. 24030462 10.1167/iovs.12-11185

[14] Montesano G, Chen A, Lu R, Lee CS, Lee AY. UWHVF: a real-world, open source dataset of perimetry tests from the Humphrey Field Analyzer at the University of Washington. *Transl Vis Sci Technol*. 2022; 11(2): 1–10. 10.1167/tvst.11.1.1 PMC8742531 34978561

[15] Huang X, Kong X, Shen Z, et al. GRAPE: a multi-modal dataset of longitudinal follow-up visual field and fundus images for glaucoma management. *Sci Data*. 2023; 10(520): 1–10. 37543686 10.1038/s41597-023-02424-4 PMC10404253

